# Truncations of the titin Z-disc predispose to a heart failure with preserved ejection phenotype in the context of pressure overload

**DOI:** 10.1371/journal.pone.0201498

**Published:** 2018-07-31

**Authors:** Lei Ye, Liping Su, Chenxu Wang, Szejie Loo, Guizhen Tee, Shihua Tan, Sandar Win Khin, Shijie Ko, Boyang Su, Stuart A. Cook

**Affiliations:** 1 National Heart Research Institute Singapore, National Heart Centre Singapore, Singapore, Singapore; 2 Duke-National University of Singapore Medical School, Singapore, Singapore; 3 National Heart and Lung Institute, Imperial College, London, United Kingdom; Augusta University Medical College of Georgia, UNITED STATES

## Abstract

Titin (TTN) Truncating variants (TTNtv) in the A-band of TTN predispose the mouse heart to systolic dysfunction when subjected to pressure-loading. However, the effects of TTNtv of the Z-disc are largely unexplored. A rat model of pressure-loaded heart is developed by trans-aortic constriction (TAC). Rats with TTNtv of the Z-disc were randomly assigned to TAC (Z-TAC) or sham-surgery (Z-Sham) and wildtype (WT) littermates served as controls (WT-TAC or WT-Sham). Left ventricular (LV) function was assessed by echocardiography. Pressure volume (PV) loops, histology and molecular profiling were performed eight months after surgery. Pressure-load by TAC increased LV mass in all cases when compared with Sham animals. Notably, systolic function was preserved in TAC animals throughout the study period, which was confirmed by terminal PV loops. Diastolic function was impaired in Z-disc TTNtv rats at baseline as compared to WT and became impaired further after TAC (dp/dt_min_, mmHg/s): Z-TAC = -3435±763, WT-TAC = -6497±1299 (p<0.01). Z-TAC animals had greater cardiac fibrosis, with elevated collagen content and decreased vascular density as compared to WT-TAC animals associated with enhanced apoptosis of myocyte and non-myocyte populations. In the context of pressure overload, Z-disc TTNtv is associated with cardiac fibrosis, diastolic dysfunction, and capillary rarefaction in the absence of overt systolic dysfunction.

## Introduction

Titin, a major myofilament of cardiac muscle, is essential for organizing muscle sarcomeres and it spans from the Z-disk to the M-band region of the cardiac sarcomere [1, 2]. It has many functions that include acting as a molecular spring, important for the passive elasticity of the heart [2]. Truncating variants in TTN (TTNtv) perturb sarcomere development [3–9] and can lead to impaired cardiac function, notably dilated cardiomyopathy (DCM) [5, 9–13].

Approximately 1% of the general population has a TTNtv, but the phenotype most commonly associated is mild and characterized by sub-clinical features of eccentric remodeling [14]. Titin truncation mutations located at A-band are a common cause of DCM [15]. In rats with TTNtv, we have shown perturbation of key signaling pathways and metabolic processes and a shift towards foetal metabolism but preserved ventricular function in the absence of injury [14]. We and others also showed that TTNtv prime the heart to fail when exposed to a second insult, such as volume overload [14] or pregnancy[16] or chemotherapy [17]. While the effects of TTNtv in the A-band in mouse predispose to systolic heart failure in the context of pressure loading [18–20], the impact of Z-disc variants are not well characterized.

Here, we used a rat model to determine whether there is an interaction between heterozygous TTNtv in the Z-disc and pressure loading induced by trans-aortic constriction (TAC). Mutant and wildtype (WT) rats were subjected to either TAC or sham surgery, and followed up by serial echocardiography. Terminal assessment of ventricular function was performed by cardiac cannulation and *ex vivo* measurements of cardiac histology and its molecular function.

## Materials and methods

### Rat pressure overload model of trans-aortic constriction (TAC)

The experimental protocol was approved by the SingHealth Institutional Animal Care and Use Committee (IACUC). All experimental and animal maintenance procedures were performed in accordance with the Animal Use Guidelines of the SingHealth IACUC and under the guidelines from Directive 2010/63/EU of the European Parliament on the protection of animals used for scientific purposes or the NIH guidelines.

Rat TTNtv model was generated by SAGE Laboratories using zinc-finger nuclease–mediated gene targeting as previously described [14]. Animals were maintained on a F344 background. Z-disc TTNtv was generated by deletion of exons 2 to 6 (5286bp deletion, coordinates 2323–7608) to introduce a frame-shift. Absence of truncated TTN protein in Z-disc TTNtv rat hearts was shown previously [14]. Male rats with Z-disc TTNtv and their wildtype littermates at 8-9-weeks of age were used for experiments. Rats were anesthetized with ketamine (100 mg/kg) and Xylazine (2.5 mg/kg) mixture. Rats were ventilated using a rodent ventilator at a respiratory rate of 90/min after intubation. The chest was shaved and disinfected with betadine and 70% alcohol. A minimally left lateral thoracotomy was performed to visualize the aortic arch, left common carotid artery and brachiocephalic artery as described [21–23]. Dissection was performed on the aortic arch between left common carotid artery and brachiocephalic artery. A sterile titanium clip (Hemoclip Plus, Weck Closure Systems, USA) loaded into the applicator was placed on the ascending aorta between the left carotid and brachiocephalic arteries. This would stop 50–60% of aortic arch flow [21–23]. The muscle layers and skin were then closed by sutures. The pressure gradient across the arch was determined by Doppler velocity using echocardiogram (Echo). The animals were kept on Baytril (15 mg/kg, *i*.*m*.) for at least three days after surgical operation to prevent infection. Ketoprofen at 2.5 mg/kg (*s*.*c*) was used for at least three days post surgery. Four animal groups were studied. They are: 1. Sham operated wildtype littermates (WT-Sham, n = 7); 2. TAC of wildtype littermates (WT-TAC, n = 11); 3. Sham operated Z-disc mutant rats (Z-Sham, n = 5); and 4. TAC of Z-disc mutant rats (Z-TAC, n = 7).

### Left ventricular function assessment by echocardiography

Transthoracic echocardiography (TTE) was performed (Vevo 2100; VisualSonics, VSI, Toronto, Canada) using a MS250 linear array transducer on rats under anesthesia (isoflurane, 1–2.5%). The rats were placed in dorsal decubitus position where their chest were shaved and a layer of acoustic coupling gel was applied to the thorax. Averages of 10 cardiac cycles of standard 2-dimensional guided m-mode short axis at mid papillary muscle level were acquired and recorded for subsequent offline analysis. Left ventricular (LV) function were determined at month-0 (as baseline), and every other month up to eight months after surgery. Measurements included LV diameter, anterior and posterior LV wall thickness at systole and diastole. LV ejection fraction (LVEF) was calculated using modified Quinone method. LV mass were calculated according to previously described method [24]. The transverse aorta was visualized with 2-dimensional imaging (S1 Fig). Guided by color flow Doppler, the peak velocity (V) distal to the contriction was measured with pulsed wave Doppler, and pressure gradient was calculated using the modified Bernoulli equation: ΔP = 4 x V^2^ [25].

### Left ventricular function assessment by pressure-volume (PV) loop

PV loop studies were performed eight months after surgery. Animals were anesthetized with ketamine (100 mg/kg) and Xylazine (2.5 mg/kg) mixture and placed in dorsal decubitus position. The neck and chest were shaved and disinfected with 70% alcohol. An incision was made on the right neck parallel to the middle line of the neck to expose and isolate the right carotid artery. A secure suture was placed around the proximal end of the artery, where a tiny incision was made near the proximal end of the artery. The catheter tip was inserted into the artery until it reached inside the ventricle and the catheter shaft was gently rotated to achieve optimal placement of the tip along the axis of the left ventricle. After stabilization for five min, the baseline PV loops was recorded at a steady state. A saline calibration (10% NaCl solution) was performed in each animal to correct volume as described [26]. For each animal, the mean of each index was calculated from 2–3 analysis that contained at least nine loops. The indices calculated were: 1) systolic indices, including stroke work (SW), ejection fraction, and dp/dt_max_, and 2) diastolic indices, including dp/dt_min_ and Tau (G).

### Tissue collection, histochemical and immuno-histochemical assessments

Under anesthesia, rat heart was arrested by direct intra-cardiac injection of 100 mg/kg KCl. Wet heart tissues were then explanted and weighed. Harvested tissues were divided into three parts: 1. embedded into paraffin for histochemical (fibrotic assessment and cardiac apoptosis assay) experiments; 2. Embedded into OCT compound for cryo-section; and 3). the remaining LV portion was used for quantitative RT-PCR, western blot, and collagen quantification.

### Cardiac fibrosis assessment

Accustain Trichrome Stains (Masson) (Sigma-Aldrich, USA) and Sirus Red/Fast Green Collagen (Chondrex, USA) staining kits were used to visualize fibrosis in rat LV. Paraffin sections with 6 μm thickness were dewaxed and stained as per supplier’s instruction [27]. To assess the severity of fibrosis in LV myocardium, pictures of Sirus Red/Fast Green stained paraffin sections were analyzed. Images of cross-sectional myocardium were processed by an in-house C++ program. Captured images were stored in Joint Photographic Expert Group (JPEG) format with 8-bit depth and the pixel value in any color channel varied from 0 to 255. To quantify fibrosis (red color) in the entire LV wall, all pixels from the image was read and categorized into three labels: collagen (red), non-collagenous proteins (green), or background after excluding staining artifacts, LV epicardium, and right ventricular wall. Pixels containing all red, green and blue values higher than 255 were considered as background and excluded from analysis. Since collagen is stained as red, a pixel was defined as red when its value was 30 times higher than the green pixel. Finally, the total number of fibrosis pixels were divided by the sum of red and green pixels to obtain the percentage of fibrosis in LV.

### Apoptosis assessment

In situ cell death detection kit (Roche, Germany) was used to determine apoptosis in tissue sections as per supplier’s instruction [28, 29]. Paraffin sections were counter-stained with α-sarcomere actin (α-SA) to visualize cardiomyocytes and DAPI to identify nuclei. The apoptotic cell number was counted in LV myocardium and presented as total TUNEL+ cardiac cells/section, while the cardiomyocyte apoptotic cell number is presented as TUNEL^+^ cardiomyocyte number/section [28, 29].

### Vascular density determination

Cryosections of 8-μm thickness were stained for CD31 and smooth muscle actin (SMA) to quantify vessel density. Blood vessel density was measured at 400X magnification in eight microscopic fields/heart after double fluorescent immunostaining for CD31 (Santa Cruz Biotechnology, Inc, USA) and SMA (Sigma Aldrich, USA) [27].

### Quantification of total collagen in rat myocardium

Collagen from the remainder LV tissue was collected using Total Collagen Assay kit from Quickzyme Biosciences (The Netherlands) as per instructions. Briefly, the tissue was loaded into 6 M HCl and incubated for 20 hours at 95°C in a thermoblock. Tissues were then homogenized and total lysates were centrifuged for 10 min at 13,000 g to collect the supernatants for analysis as per instruction.

### Quantitative RT-PCR analysis of rat cardiac tissue

To determine fibrosis and cardiac injury, total RNA was isolated from rats at eight months after surgery as described [30, 31] The gene expression level of collagen type I alpha 1 chain (Collagen-I), collagen type III alpha 1 chain (Collagen-III), natriuretic peptide B (BNP), and natriuretic peptide A (ANP) were quantified after normalized with GAPDH. Primers used were: 1. Collagen-I (98 bp): sense ACTGGTACATCAGCCCAAAC and antisense GGAACCTTCGCTTCCATACTC; 2. Collagen-III (120 bp): sense CTGAACTCAAGAGCGGAGAATAC and antisense: CAGTCATGGGACTGGCATTTA; 3. BNP (85 bp): sense CTAGCCAGTCTCC AGAACAATC and antisense AGCTGTCTCTGAGCCATTTC; 4. ANP (106 bp) sense: TCCGATAGATCTGCCCTCTT and antisense: CTCCAATCCTGTCAATCCTACC; 5. GAPDH (105 bp) sense: ACTCCCATTCTTCCACCTTTG and antisense: CCCTGTTGCTGTAGCCATATT. Gene expression level was compared with that of WT sham group (considered as 100%) and expressed as either fold increase or decrease.

### Western blot analysis

To determine the caspase 3 activity and Bax protein expression level, total rat cardiac protein from the LV tissue was isolated using PhosphoSafe™ Extraction Reagent (Merck, Germany) and protein concentration was determined using Bradford reagent (Bio-Rad Laboratories, USA) as per instruction. Proteins (20 μg/ sample) were separated on SDS-polyacrylamide gel and transferred onto nitrocellulose membrane. After blocking with 5% non-fat milk in Tris-buffered saline Tween-20 buffer (TBST; 25 mM Tris, pH 7.5, 150 mM NaCl, and 0.1% Tween-20), the membranes were incubated with either 1: 500 diluted rabbit anti cleaved caspase 3 or 1: 2000 rabbit anti Bax (all purchased from Cell Signaling, USA) for overnight at 4°C. Anti-rabbit IgG conjugated with HRP (dilution: 1: 2,000) was used for antibody detection. Binding of the specific antibody was visualized using Super Signal Chemiluminescent Substrate kit (Pierce, USA) and visualized by ChemiDocTM MP Imaging System (Bio-Lab, USA) and quantified using Image Lab 5.1 software (Bio-Lab, USA). The optical intensity of the band was quantified and expressed as percentage of GAPDH (regarded as 100%).

### Statistical analyses

Statistical analyses were performed using SPSS (version 20.0). All data were presented as mean ± standard deviation (SD). Comparisons among groups for significance were conducted using one-way analysis of variance (ANOVA), where p-value <0.05 was considered statistically significant. Post-hoc analyses were performed using Tukey’s Test with a significance level of 5%.

## Results

### LV function by echocardiography

Heart function and morphology was assessed by serial echocardiograph. Surprisingly, unlike the A-band TTNtv mouse [14] systolic function remained preserved for a prolonged period in the Z-TAC rats (S1 Table) despite sustained and marked pressure loading (S1 Fig). LV function trended downwards in both TAC groups at 8 months of follow-up, at which time echocardiograms were performed in detail (Table 1). Then, hearts were harvested to assess functional and molecular factors.

**Table 1:** Cardiac function at 8 months after surgery as assessed by echocardiogram.

	WT-Sham (n = 6)	Z-Sham (n = 5)	WT-TAC (n = 11)	Z-TAC (n = 7)
Body weight (g)	435.3±20.3	413.6±23.2	445±26.7	426±13.9
Pressure gradient (mmHg)	2.2±0.5	2.5±0.5	51.7±13.4^&^	54±13.2^&^
LVEF (%)	79.8±3.4	81.3±3.4	70.8±6.2	71.6±6.5
Anted (mm)	1.99±0.15	2.0±0.07	2.11 ±0.12	2.05 ±0.10
Antes (mm)	3.64±0.26	3.52±0.13	3.46±0.26	3.45±0.17
PLVWed (mm)	1.91±0.18	1.85±0.02	2.13±0.1*^,^^##^	2.2±0.14**^,^^###^
PLVWes (mm)	3.24±0.23	3.3±0.11	3.37±0.24	3.31±0.19
LV mass (mg)	1024.7±62.9	992.6±87.1	1246.9±78.1^&^	1258.3±94.1^&^

LVEF: left ventricular ejection fraction

Anted: anterior left ventricular wall thickness at end of diastole

Antes: anterior left ventricular wall thickness at end of systole

PLVWed: posterior left ventricular wall thickness at end of diastole

PLVWes: posterior left ventricular wall thickness at end of systole

LV: left ventricular.

*: compared with WT-Sham: p<0.05

**: compared with WT-Sham: p<0.01.

##: compared with Z-Sham: p<0.01

###: compared with Z-Sham: p<0.001

&: compared with WT-Sham and Z-Sham: p<0.001.

### TAC causes diastolic dysfunction in rats with TTNtv

**Fig 1:**
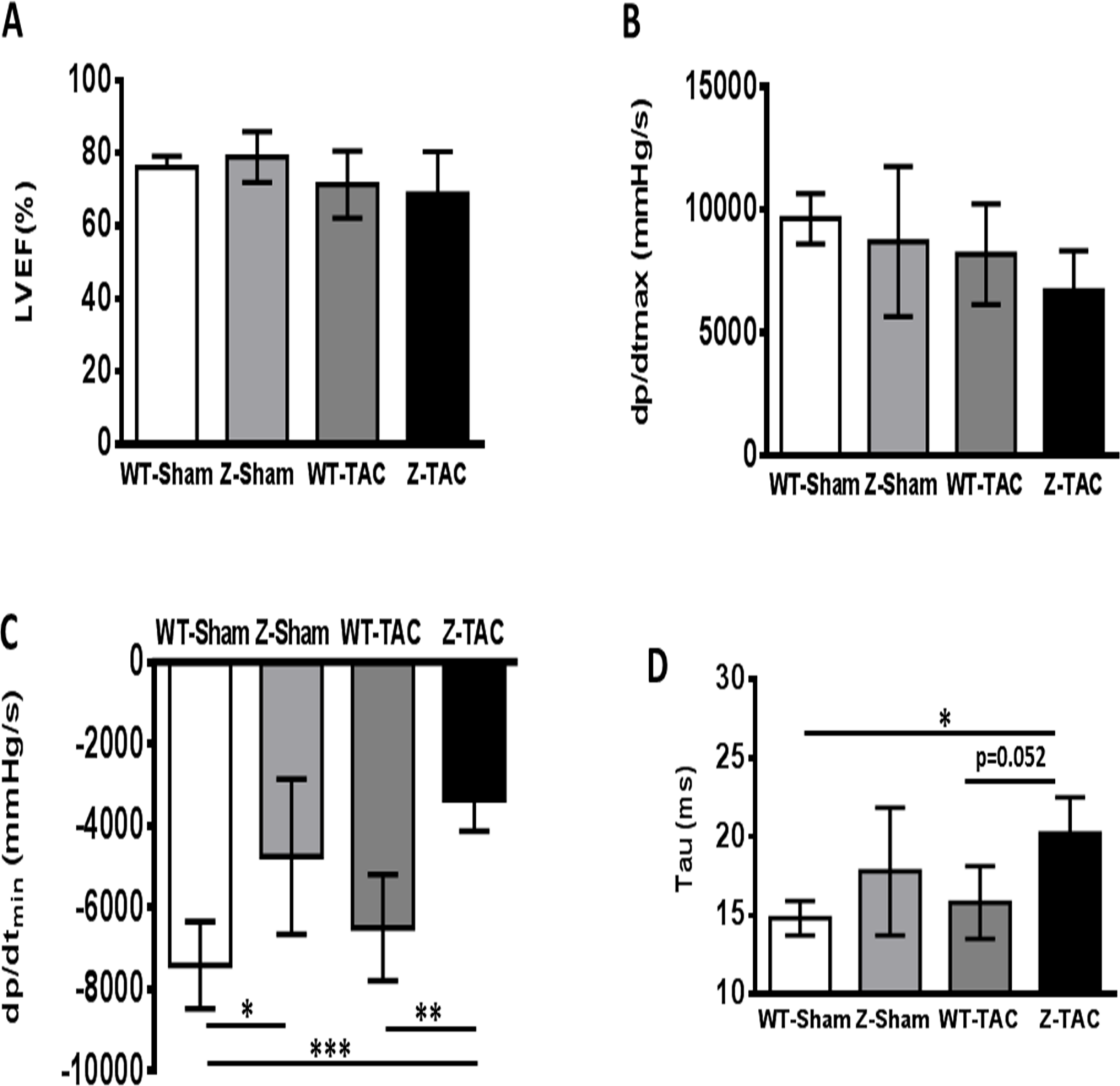
Rat LV function assessed by PV loop. LV systolic function indexes: **(A)** Left ventricular ejection fraction (LVEF), **(B)** dp/dt_max_. LV diastolic function indexes: **(C)** dp/dt_min_ and **(D)** Tau. (*: p<0.05; **: p<0.01; ***: p<0.001.

PV loop studies were performed in rats as a terminal procedure. EF and stroke work in WT-TAC and Z-TAC animals were similar to WT-Sham and Z-Sham groups (Fig 1A and S2 Fig). There was a non-significant downward trend in contraction rate (dp/dt_max_) in the Z-TAC group (Fig 1B). Overall, the PV loop data confirmed that the overall systolic function in the Z-TAC rats was largely preserved as compared to other experimental groups after 8 months of TAC.

Diastolic function, as assessed by dp/dt_min_ (LV relaxation rate), was impaired at baseline (Z-Sham) and after TAC (Z-TAC) in mutant rats as compared to WT-Sham and WT-TAC groups, with Z-TAC rats exhibiting the worst diastolic function (Fig 1C). The Tau (relaxation time constant) of the Z-TAC group was significantly higher compared to the WT-Sham group (p<0.05) and was trending up compared to WT-TAC (p = 0.052) (Fig 1D). These data show that Z-disc TTNtv in the rat causes diastolic dysfunction at baseline and that this effect is exacerbated by pressure overload.

### Increased myocardial fibrosis after TAC in rats with TTNtv

The ratio of wet heart weight/body weight (mg/g) was increased by TAC (WT-Sham 2.52±0.11 vs WT-TAC 2.86±0.22 (p<0.05); Z-Sham, 2.61±0.29 vs Z-TAC, 3.13 ± 0.08 (p<0.01)).

**Fig 2:**
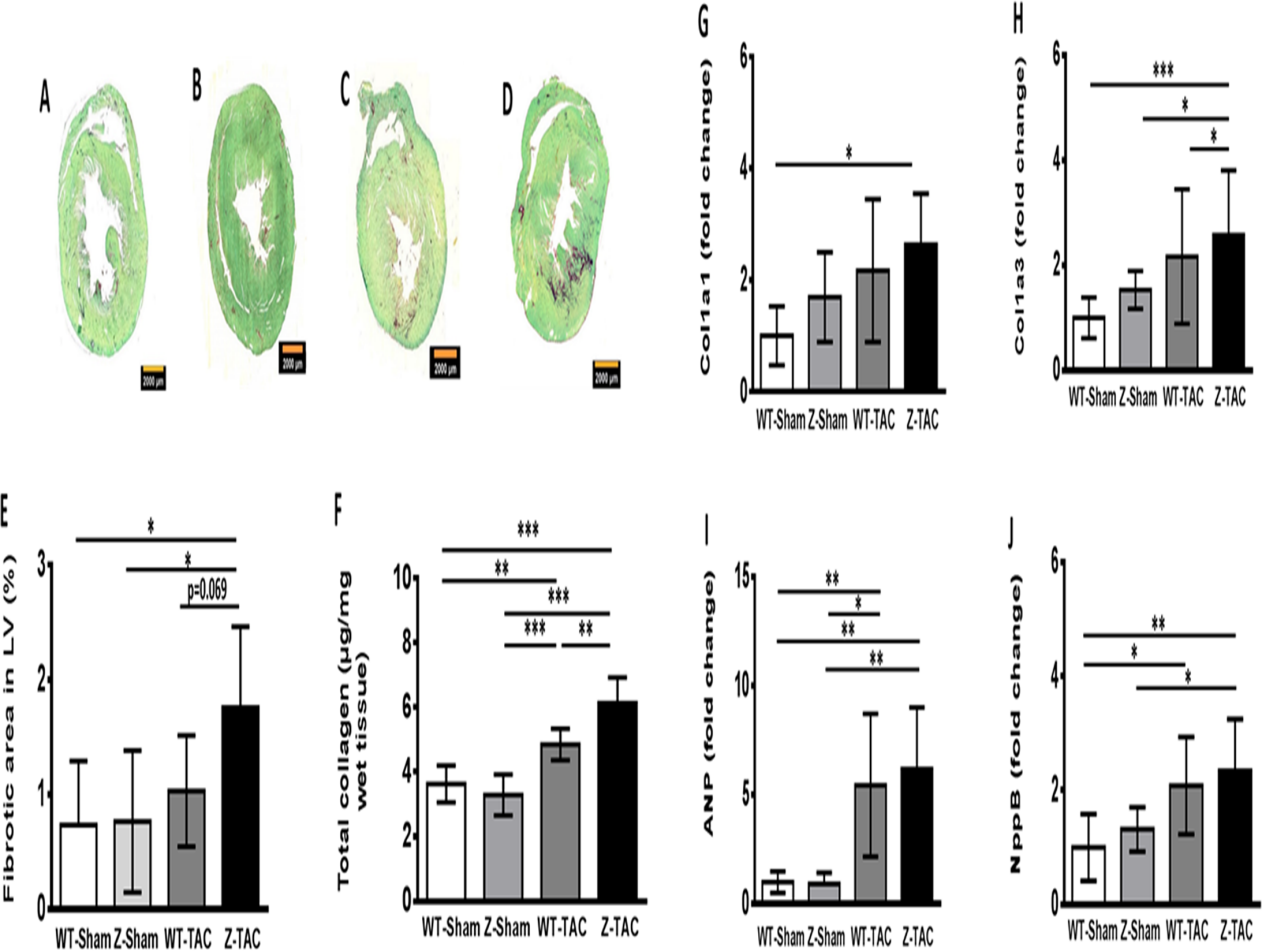
Rat heart weight/bodyweight eight months post-surgery. Representative pictures of Sirus Red/Fast Green Collage staining to visualize fibrosis in rat heart of WT-Sham **(A)**, Z-Sham **(B)**, WT-TAC **(C)**, and Z-TAC **(D)** groups. **(E)** Quantification of LV fibrosis using Sirus red/Fast green stained rat cardiac tissue sections. **(F)** Quantification of total collagen in rat myocardium. Gene expression levels of rat collagen type I alpha 1 chain (Col1a1) **(G)**, collagen type III alpha 1 chain (Col3a1) **(H)**, atrial natriuretic peptide (ANP) **(I)**, and natriuretic peptide B (NppB) **(J)** in rat myocardium. (*: p<0.05; **: p<0.01; ***: p<0.001. Bar = 2000 μm).

Sirus Red/Fast Green Collagen and Masson trichrome staining showed significantly increased myocardial fibrosis in Z-TAC group (Fig 2A–2E and S3 Fig). Molecular quantification of collagen content confirmed that both TAC groups had significantly increased collagen content in LV myocardium with Z-TAC group having significantly higher amounts than the other three groups (Fig 2F).

Gene expression levels of collagen type I alpha 1 chain and collagen type III alpha 1 chain were mildly elevated in Z-Sham at baseline and in both TAC groups with highest levels in the Z-TAC (Fig 2G & 2H). Similarly, ANP and NppB gene expression levels were increased in both TAC groups (Fig 2I & 2J).

### Capillary rarefaction in Z-disc TTNtv rat heart after TAC

**Fig 3:**
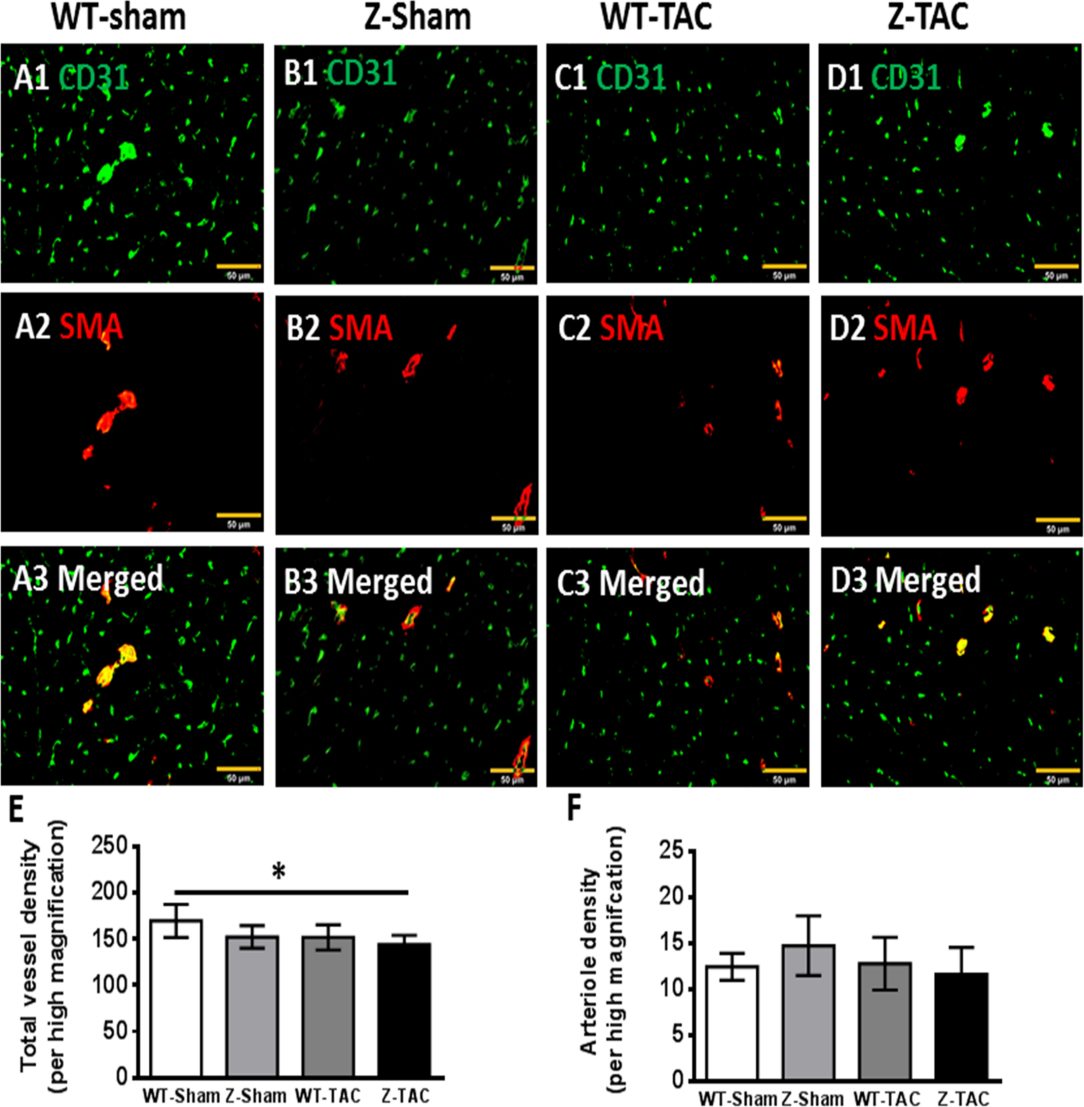
Vascular density and arteriole density were evaluated in cryo-sections eight months after TAC from the **(A1-A3)** WT-Sham, **(B1-B3)** Z-Sham, **(C1-C3)** WT-TAC, and **(D1-D3)** Z-TAC groups via immunofluorescent staining for CD31 and SMA. **(E)** Total vessel density was determined by counting CD31+ vascular structures, and **(F)** arteriole density was determined by counting vascular structure that co-expressed CD31 and SMA. (*: p<0.05. Bar = 50 μm).

The total vessel density based on CD31 fluorescence immunostaining was reduced in Z-Sham, WT-TAC and Z-TAC groups, especially so in Z-TAC rats, which had significantly lower vessel density as compared with WT-Sham group (Fig 3A1–3D1 & 3E). Arteriole density based on the co-immunostaining for CD31 and SMA (Fig 3A2–3D2 & 3A3–3D3) was similar in all groups (Fig 3F).

### Apoptosis in TTNtv rat hearts after TAC

**Fig 4:**
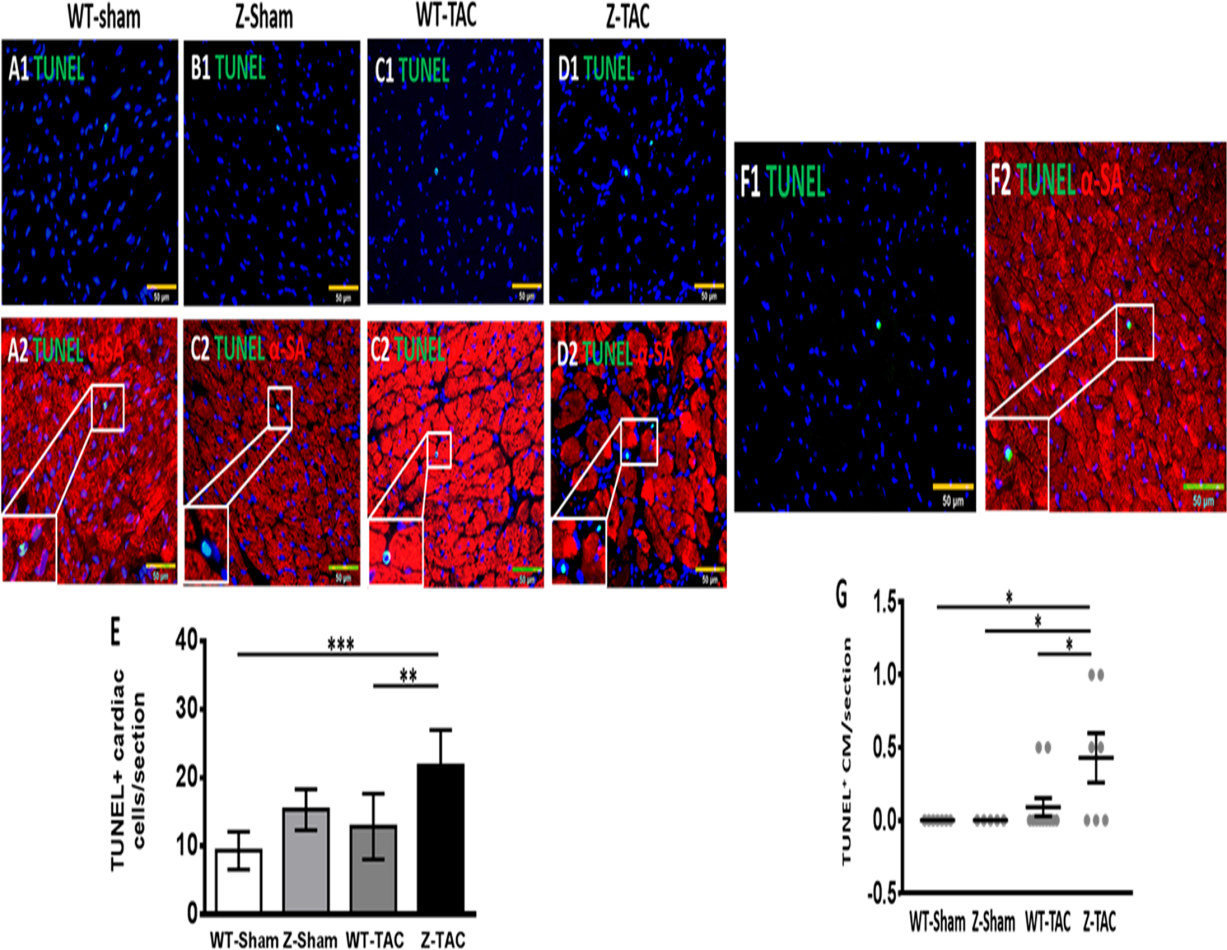
TUNEL staining of rat cardiac tissue to identify apoptotic cells. TUNEL^+^ nuclei in WT-Sham **(A1)**, Z-Sham **(B1)**, WT-TAC **(C1)**, and Z-TAC **(D1)** rat hearts. The same sections were count-stained with α-sarcomere actin (α-SA) to show the localization of TUNEL+ nuclei in rat cardiac tissue **(A2-D2)**. **(E)** Quantification of TUNEL^+^ cardiac cells per LV section. Representative pictures of a TUNEL^+^ nucleus **(F1)** located in a cardiomyocyte that was stained for α-SA expression **(F2)**. **(G)** Quantification of TUNEL^+^ cardiomyocytes per LV section. (*: p<0.05; **: p<0.01; ***: p<0.001. Bar = 50 μm).

TUNEL staining showed that pressure overload increased apoptotic cells in both TAC groups, with significantly higher levels in the Z-TAC group (21.8 ± 2.0/section) when compared to the WT groups (Fig 4A–4E). Apoptotic cardiomyocytes were found in the LV of 4 out of 7 rats in Z-TAC group (Fig 4F), while 2 of 11 rats in the WT-TAC group had apoptotic cardiomyocytes in the LV (Fig 4G). There were no apoptotic cardiomyocytes found in WT-Sham and Z-Sham groups.

**Fig 5:**
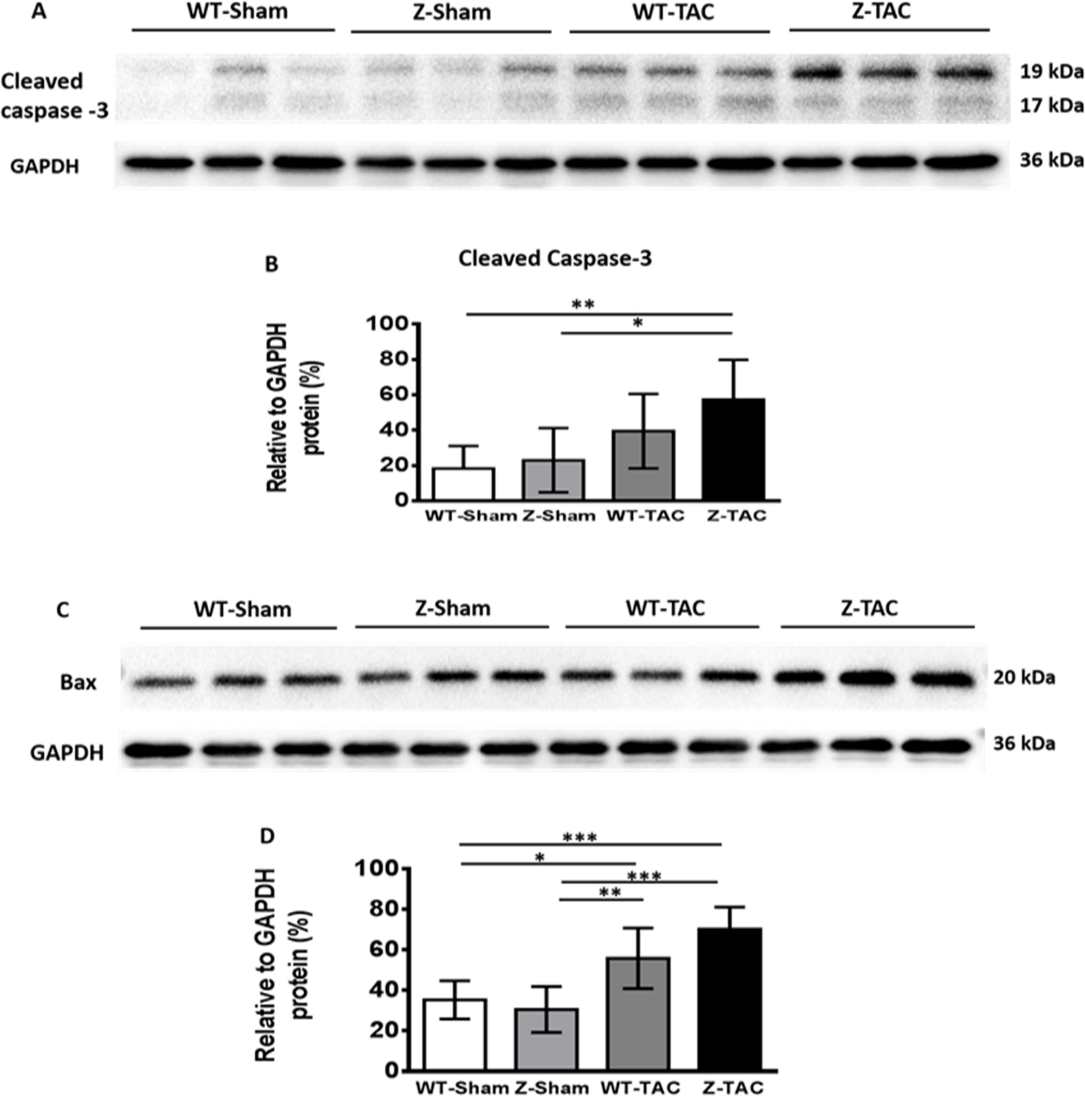
Western blot analysis of rat myocardium to determine the activity of caspase 3 and Bax. **(A)** Image of cleavage caspase 3 protein expression. **(B)** Quantification of cleaved caspase 3 protein expression after normalized with GAPDH protein expression level. **(C)** Image of Bax protein expression. **(D)** Quantification of Bax protein expression after normalized with GAPDH protein expression level. (*: p<0.05).

Protein expression levels of cleaved caspase 3 in the Z-TAC group by Western blot was significantly higher compared to the WT-Sham and Z-Sham groups (Fig 5A & 5B). Bax increased significantly in both TAC groups when compared to the WT-Sham and Z-Sham groups (Fig 5A–5D).

## Discussion

Previous studies in the mouse have focused on A-band TTNtv [18–20] and shown impairment of LVEF and diffuse myocardial fibrosis when mice are subjected to pharmacological stress [19] or pressure overload [18]. Here we studied the effect of Z-disc TTNtv in the rat and its interaction with pressure loading and found an exaggerated phenotype of cardiac fibrosis, apoptosis, and vascular rarefaction similar to that seen in the heart failure with preserved ejection fraction (HFpEF). Z-disc TTNtv rats were still at compensated stage at 8 months’ after TAC as evidenced by preserved systolic function and no notably dilated cardiomyopathy was observed.

The Z-disc senses, integrates, and transduces biomechanical stress signals [32]. Mouse knock-out studies of Z-disc proteins, including muscle LIM protein [33], calsarcin-1 [34], and the costameric protein melusin [35] have shown that defects of Z-disc proteins are associated with heart failure phenotypes. Here, in a rat model of Z-disc TTNtv we observed a predominant diastolic heart failure phenotype.

We found significantly increased non-myocyte apoptosis in hearts of Z-TAC rats with compensated hypertrophy. Non-myocytes have been reported as the predominant apoptotic cell type in compensated pressure overload, whereas myocyte apoptosis are mainly found in decompensated stages associated with systolic dysfunction [36–39]. Limited numbers of apoptotic myocytes were found in both TAC groups. We speculate that the increased non-myocyte apoptosis may play a role in the fibrosis and diastolic phenotypes we observed.

Microvascular rarefaction is a feature of structural change in HFpEF[40], which was also observed in the current study. It’s unknown that apoptosis of non-myocyte in Z-TAC rat can cause capillary rarefaction or not. However, cardiac hypertrophy can activate NF-ĸB, which may cause capillary rarefaction through regulation of matrix metalloproteinase (MMP) activity [41–43].

Another hallmark feature of cardiac remodeling is the deposition of excessive extracellular matrix [44, 45]. We found that Z-disc TTNtv results in an increase in ventricular fibrosis in the context of pressure overload. Increased fibrosis causes myocardial stiffness, which can contribute to diastolic dysfunction [46, 47]. Our PV loop data showed significantly impaired LV relaxation in Z-disc mutant rats at baseline and even more so after TAC, which may relate to properties intrinsic to the myocyte at baseline and to greater fibrosis after TAC.

Our study has limitations. We did not assess cardiac inflammation or plasma cytokine levels, such as TNF-α and IL6, which contributes to changes in the extracellular matrix in HFpEF. We also did not measure reactive oxygen species (ROS) production, which can trigger cardiac myocyte apoptosis [48, 49]. Nor did we measure individual myocyte function that may be affected by mutation of the titin Z-disc.

In conclusion, TTNtv in the Z-disc interact with pressure overload and result in a cardiac phenotype defined by fibrosis, reduced vascular density, non-myocyte apoptosis and impaired diastolic function with preserved EF.

## Supporting information

S1 Fig Representation of color flow and pulsed wave Doppler images of the aortic arch in a WT-Sham and a WT-TAC rat. Typical color flow Doppler depicting normal aortic flow in a WT-Sham rat (**A**). Sample volume with proper angle correction was set approximately 1mm proximal from LCCA to obtain aortic pulse wave velocity image (**B**). Color aliasing (white-color arrow) corresponding to the stenosis (**C**) was visualized in a TAC rat, and was used to guide the placement of sample volume to obtain highest post constriction pulsed wave velocity (**D).** (IA = Innominate artery; LCCA = Left common carotid artery; LSA = Left subclavian artery).
(TIFF)

S2 Fig Stroke work (SW) as assessed by PV loop. (WT-Sham = 5, Z-Sham = 5, WT-TAC = 6, and Z-TAC = 5).
(TIFF)

S3 Fig Representative pictures of Masson trichrome staining to visualize fibrosis in rat heart of WT-Sham **(A)**, Z-Sham **(B)**, WT-TAC **(C)**, and Z-TAC **(D)** groups.
(TIFF)

S1 Table TAC rat cardiac function assessed by echocardiogram. (PDF)
