# Supplementary figures and images for: Truncations of the titin Z-disc predispose to a heart failure with preserved ejection phenotype in the context of pressure overload
Source: PLoS One. 2018 Jul 31;13(7):e0201498. doi: 10.1371/journal.pone.0201498 (PMC6067738; doi:10.1371/journal.pone.0201498)

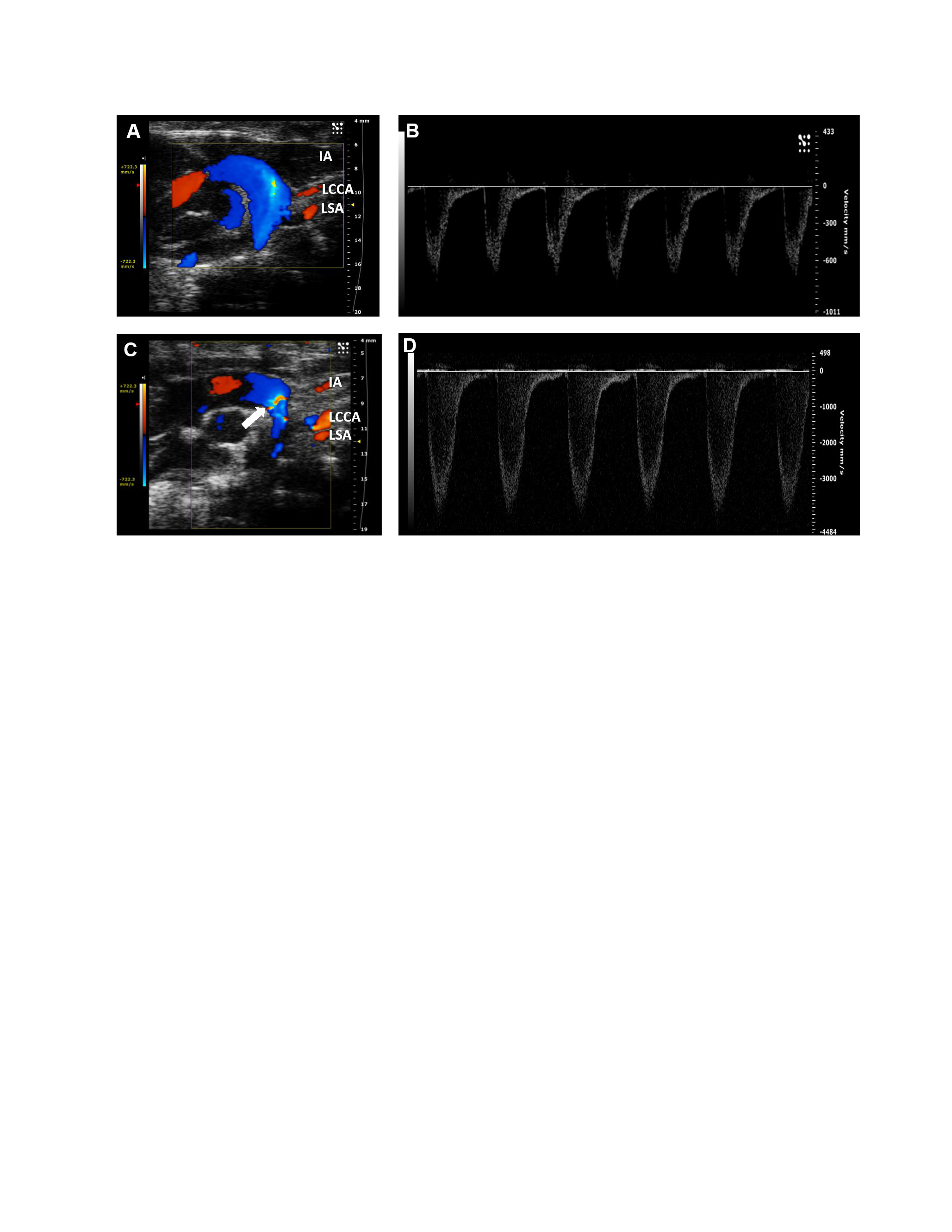

Supplement: S1 Fig — Typical color flow Doppler depicting normal aortic flow in a WT-Sham rat (A). Sample volume with proper angle correction was set approximately 1mm proximal from LCCA to obtain aortic pulse wave velocity image (B). Color aliasing (white-color arrow) corresponding to the stenosis (C) was visualized in a TAC rat, and was used to guide the placement of sample volume to obtain highest post constriction pulsed wave velocity (D). (IA = Innominate artery; LCCA = Left common carotid artery; LSA = Left subclavian artery). (TIFF) [file pone.0201498.s001.tiff]

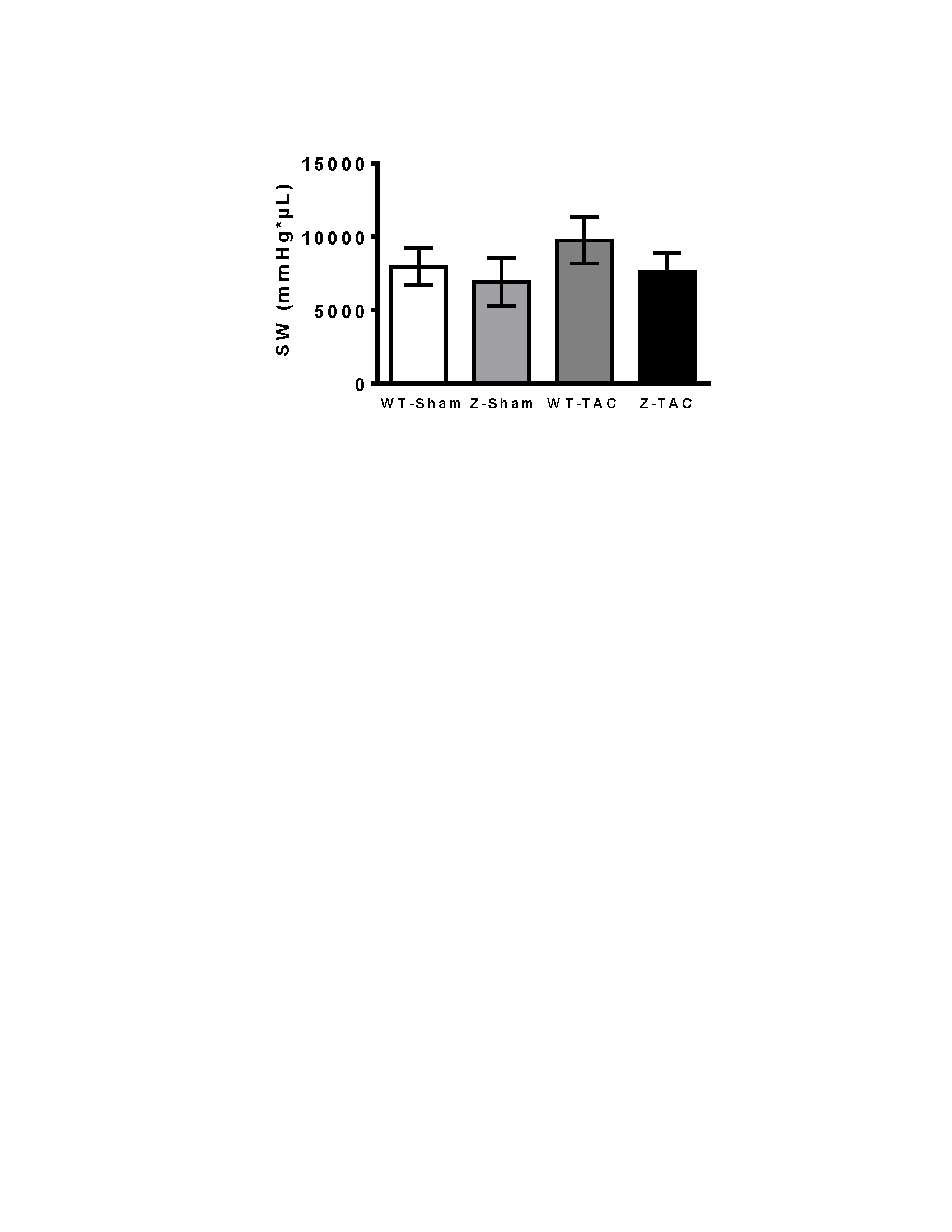

Supplement: S2 Fig — (WT-Sham = 5, Z-Sham = 5, WT-TAC = 6, and Z-TAC = 5). (TIFF) [file pone.0201498.s002.tiff]

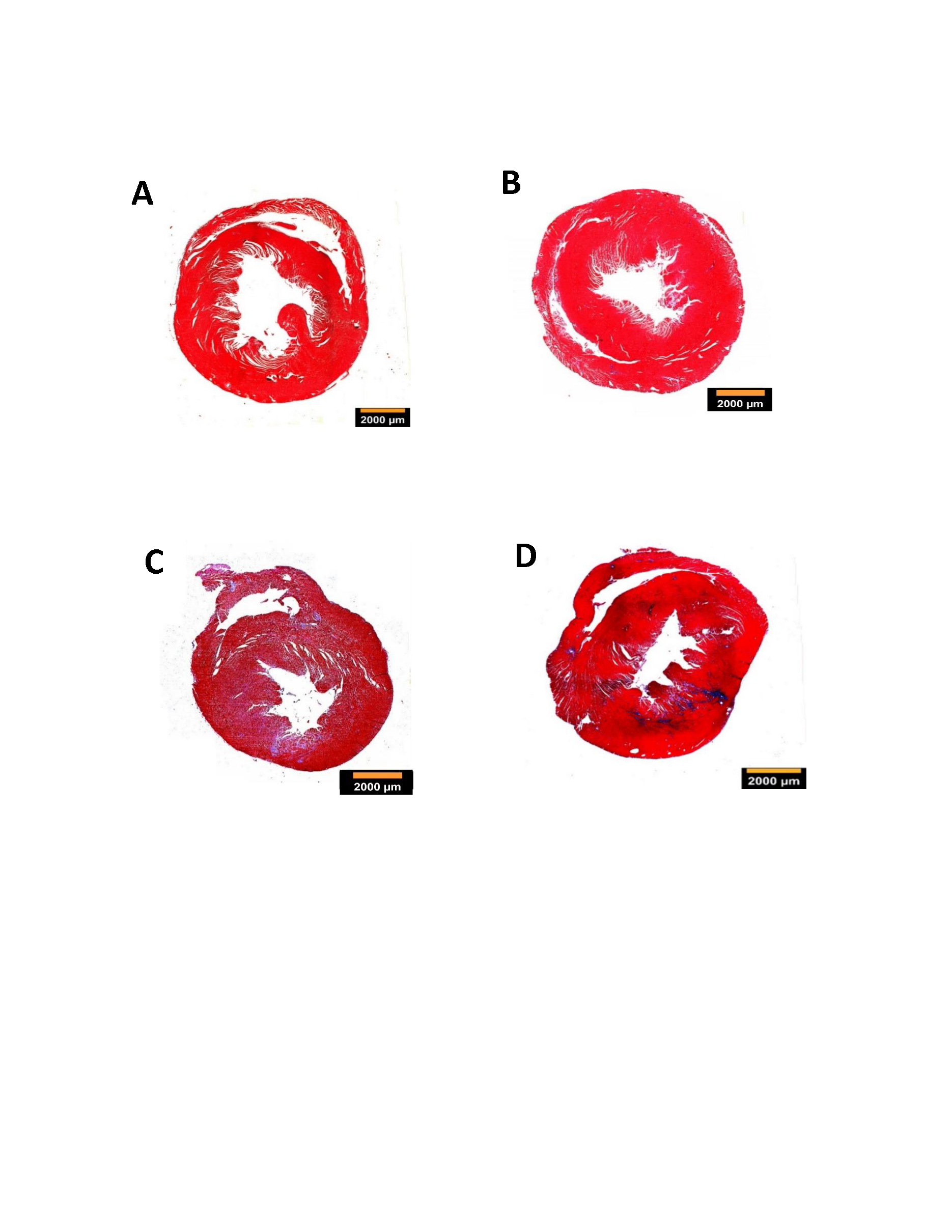

Supplement: S3 Fig — Representative pictures of Masson trichrome staining to visualize fibrosis in rat heart of WT-Sham (A), Z-Sham (B), WT-TAC (C), and Z-TAC (D) groups. (TIFF) [file pone.0201498.s003.tiff]
