# Supplementary material for: Truncations of the titin Z-disc predispose to a heart failure with preserved ejection phenotype in the context of pressure overload
Source: PLoS One. 2018 Jul 31;13(7):e0201498. doi: 10.1371/journal.pone.0201498 (PMC6067738; doi:10.1371/journal.pone.0201498)
Supplement: S1 Table — (PDF) [file pone.0201498.s004.pdf]

S1 Table. TAC rat cardiac function assessed by echocardiogram

Pressure gradient (mmHg)	Month 0	Month 2	Month 4	Month 6	Month 8
WT-Sham (n=6)	3.4±0.7	2.7±1.1	2.6±0.5	2±0.2	2.2±0.5
WT-TAC (n=11)	3.1±0.8	47.8±7.4*	53.9±13.6*	50.7±20.2*	51.7±13.4
Z-TAC (n=7)	3.80±0.9	48.9±7.6*	51.8±11.6*	42.7±13.3*	54±13.2
**EF%**					
WT-Sham (n=6)	80.3±3.7	82.4±3.7	76.1±3.6	75.5±4	79.8±3.4
WT-TAC (n=11)	82.1±6.8	82.3±4.3	79.9±7	73.3±4.3	70.8±6.2
Z-TAC (n=7)	80.2±4	79.9±6.8	72.6±8.3	66.9±7.7	71.6±6.5
**LV mass (mg)**					
WT-Sham (n=6)	769.9±77.1	910.2±49.2	962.7±69.9	997.8±63.6	1024.7±62.9
WT-TAC (n=11)	730.11±51.7	1074.5±75.8*	1121.3±78.4*	1161.1±72*	1246.9±78.1*
Z-TAC (n=7)	741.1±51.6	1058.±81.2*	1148.3±85.7*	1123.8±148.6	1258.3±94.1*

***: compared with WT-sham: p<0.05.**
